# Can Targeted Poverty Alleviation Program Reduce Depression? Evidence From China

**DOI:** 10.3389/ijph.2024.1607106

**Published:** 2024-08-30

**Authors:** Ziying Yang, Chang Xiong, Manping Tang

**Affiliations:** ^1^ School of Finance, Southwestern University of Finance and Economics, Chengdu, Sichuan, China; ^2^ Institute of Chinese Financial Studies, Southwestern University of Finance and Economics, Chengdu, China; ^3^ College of Management, Sichuan Agricultural University, Chengdu, Sichuan, China

**Keywords:** targeted poverty alleviation, depression, mental health, mechanisms, difference-in-difference model

## Abstract

**Objectives:**

This study aimed to examine whether China’s Targeted Poverty Alleviation (TPA) program mitigates depression and explores the mechanisms through which the TPA program affects individuals’ depression.

**Methods:**

Using the data from the China Family Panel Studies (CFPS) survey from 2012 to 2020, we employ a Difference-in-Difference model to analyze the effect of the TPA program on individuals’ depression levels.

**Results:**

Our findings indicate that the TPA program reduces depression scores by 0.116 points, accounting for 6.82% of the standard deviation of depression scores. Further analyses indicate that these effects are mediated through improvements in local medical conditions, reductions in household healthcare spending, increases in household entertainment expenditures, and greater likelihood of living in family.

**Conclusion:**

This study showed that the TPA program significantly mitigates individuals’ depression levels. The possible channels include (1) improving local medical conditions, (2) cutting down household healthcare spending, (3) increasing household entertainment expenses, and (4) increasing the likelihood of living in family.

## Introduction

Research indicates that poverty is associated with mental health issues such as depression [1, 2]. However, existing literature on poverty alleviation has predominantly focused on economic outcomes such as income [3, 4], consumption [5, 6], and access to credit [7]. Little attention has been given to how poverty alleviation programs impact mental health. Given the significant role of mental health in overall wellbeing and the notable success of China’s Targeted Poverty Alleviation (TPA) program (See more details about the program in the Supplementary File S1), this study aims to fill this gap by investigating whether and how the TPA program mitigates depression.

We hypothesize that the TPA program alleviates depression through several mechanisms. Introduced by President Xi Jinping in November 2013 and fully implemented by 2015, the TPA program targeted households with *per capita* incomes below the national poverty line, with the goal of lifting all such households out of poverty by 2020. The program contributes to reducing depression by increasing household income, thereby alleviating financial stress and allowing for higher expenditures on entertainment [8, 9]. Moreover, by enrolling the poor in the social security system and improving local healthcare infrastructure, the TPA program reduces healthcare expenditures and enhances access to medical treatment, thereby further alleviating financial stress. Additionally, the TPA program helps create employment opportunities for those households covered by the TPA program in their hometowns, so that individuals in these households can find jobs in their hometown. As a result, they can live with family, which is associated with family happiness and lower levels of depression [10].

To empirically examine the impact of the TPA program on depression levels, we utilize data from the China Family Panel Studies (CFPS) survey conducted by the Institute of Social Science Survey (ISSS) at Peking University from 2012 to 2020. Employing a Difference-in-Difference (DID) model, our analysis demonstrates a statistically significant reduction in depression levels attributable to the TPA program. Specifically, the TPA program reduces depression scores by 0.116 points, accounting for 6.82% of the standard deviation of depression scores. Further analyses indicate that these effects are mediated through improvements in local medical conditions, reductions in household healthcare spending, increases in household entertainment expenditures, and greater likelihood of living in family.

This study contributes to the literature in several ways. Firstly, it provides a comprehensive evaluation of the effects of China’s TPA program, expanding beyond its economic impacts. While previous studies have highlighted income increases [11, 12], consumption enhancements [6], and improved access to credit [7], our study is among the first to examine its effects on depression among the poor in China.

Secondly, our research contributes to the broader literature on poverty alleviation programs and mental health. Existing studies have explored the impact of poverty alleviation efforts on mental health through different lenses, such as microfinance-based programs and monetary poverty alleviation programs for children and adolescents [13, 14]. By identifying specific mechanisms through which the TPA program affects depression, our study enriches this literature and provides insights applicable to similar programs globally.

Finally, our findings hold practical implications for policymakers worldwide, offering insights into designing effective anti-poverty policies that also address mental health issues. Poverty has long been a global problem [15]. China has nearly one-fifth of the world’s population. With the TPA program, China successfully lifted all rural poor households out of poverty by 2020. China eradicated extreme poverty 10 years ahead of the first target of the UN 20230 Agenda for Sustainable Development [16]. This is a huge success against poverty, which can provide for other countries in eradicating poverty. For example, Pakistan and some African countries are considering adopting China’s poverty alleviation model. Our study underscores the importance of integrating mental health considerations into poverty alleviation strategies, thereby contributing to broader efforts aimed at poverty reduction and enhancing wellbeing globally.

## Methods

### Data and Sample

The dataset for this study is derived from the China Family Panel Studies (CFPS) survey from 2012 to 2020, conducted by Institute of Social Science Survey (ISSS) at Peking University. This longitudinal survey was conducted biennially. To ensure sample representativeness, CFPS employs a multi-stage probability sampling method using implicit stratification. This approach is akin to methods used in well-known surveys such as the Panel Study of Income Dynamics (PSID), the National Longitudinal Surveys of Youth (NLSY), and the Health and Retirement Study (HRS) in the United States. Previous research has established that the CFPS survey provides a near-nationally representative sample [17].

The data set includes: (1) household information, such as household income, assets, expenses, and family size; (2) individual’s demographic information, such as age, marriage status, health, education, social status, and living in family or not; (3) other individual’s information, such as depression conditions, and perception of local medical condition. We exclude observations with missing values for the key variables. Finally, our sample consists of 129,713 individual-year observations, covering 13,405 households in 551 counties from 31 provinces in China.

### Variable Definitions and Summary Statistics

The definitions of the variables in this study are presented in Table 1. Specifically, we construct measurement of individual depression based on the Center for Epidemiological Studies Depression (CESD) scales. The CFPS used CESD-8: (1) I am in a low spirit. (2) I find it difficult to do anything. (3) I cannot sleep well. (4) I feel happy. (5) I feel lonely. (6) I have a happy life. (7) I feel sad. (8) I feel that I cannot continue with my life. Respondents were asked to pick one of the following options for each question: 1. Never (less than 1 day), 2. Sometimes (1-2 days), 3. Often (3-4 days, and 4. Most of the time (5–7 days). CESD-8 has been widely used to assess depressive symptoms [18]. Unlike other questions, in questions (4) and (6), the larger the score is, the less depressive the individual is. To address the inconsistency, we first reverse the meaning of values in questions (4) and (6). Then, we compute the total score of the 8 questions. Finally, we rescale the score by min-max normalization and then multiply by 10. The final score measures an individual’s depression level ( Depression ). Consequently, the larger the score is, the more depressive the individual is.

**TABLE 1:** Variable definitions (China, 2024).

Variables	Description
Depression	Depression level of an individual
Treat	Defined as 1 if an individual’s household is included in the TPA program, and 0 otherwise
Post	Defined as 1 for subsequent years after 2015, and 0 otherwise
Log (Income)	The natural logarithm of household total income (RMB)
Log (Debts)	The natural logarithm of household total debts (RMB)
Family size	The family size of the household
Age	The individual’s age
Marriage	The individual’s marriage status, denoted as 1 for married and 0 otherwise
Health	The health level of the individual
Education	The individual’s years of schooling
Social status	The social status of the individual in local place, ranging from very low to very high
Medical conditions	The individual’s perception of local medical level, ranging from “1: Very bad” to “5: Very good”
Healthcare spending	The sum of household total medical expenditure and expenditure on fitness
Entertainment expenses	The sum of household total expenditure on entertainment and travel expenses
Living in family	Defined as 1 if an individual lived in family, and 0 otherwise

In addition, following the literature [19], we construct treatment status based on household income *per capita* and China’s income poverty lines. The Chinese government implemented the TPA program at the end of 2014, and those households whose income *per capita* is below the national income poverty line were covered by the program. Thus, the treatment variable Treat is defined as 1 if an individual’s household income *per capita* is below the poverty line in either 2014 CFPS or 2016 CFPS, and 0 otherwise. We also introduce other measures of treatment status in the Section of robustness checks. Finally, given that the TPA program started at the end of 2014, Post is defined as 1 for subsequent years after 2015, and 0 otherwise.

Table 2 presents the summary statistics of variables. All continuous variables are winsorized at 1% and 99% to exclude the effect of outliers. The mean of Depression is 2.186 out of 10 with a standard deviation of 1.783. The mean of Treat is 0.190, suggesting that 19% of the households in the sample were covered by the TPA program. The average $\mathrm{Log}\left(Income\right)$ is 10.423, which is equivalent to 59,137 RMB Yuan.

**TABLE 2:** Summary statistics (China, 2024).

Panel A: Household-year level characteristics								
Variable	N	Mean	S.D.	Min	P25	P50	P75	Max
Treat	55,057	0.190	0.392	0	0	0	0	1
Log(Income)	55,057	10.423	1.154	6.553	9.904	10.597	11.156	12.766
Log(Debts)	55,057	3.485	5.116	0	0	0	9.904	13.63
Family size	55,057	3.635	1.804	1	2	3	5	10

Panel B: Individual-year level characteristics								
Variable	N	Mean	S.D.	Min	P25	P50	P75	Max
Depression	0.833	1.950	1.783	0			3.297	10
Age	129,713	45.666	16.610	16	32	46	59	81
Marriage	129,713	0.787	0.410	0	1	1	1	1
Health	129,713	2.986	1.219	1	2	3	4	5
Education	129,713	7.810	4.788	0	6	9	12	16
Social status	129,713	2.899	1.050	1	2	3	3	5

### Empirical Model

To examine the impact of the TPA program on individual’s depression, we set a Difference-in-Difference (DID) model as follows:

$$Depression_{ijt}=\alpha +\beta Treat_{j}\times Post_{t}+\gamma X_{ijt}+u_{j}+\lambda_{t}+\varepsilon_{ijt}$$ (1)

where i , j , and t index individual, household, and year, respectively. $Depression_{ijt}$ is the depressive level for individual i in household j in year t . $Treat_{j}$ is an indicator variable which is defined as 1 if household j was included by the TPA program, and 0 otherwise. $Post_{t}$ is a dummy variable, which equals 1 for subsequent year after 2015 and 0 otherwise. $X_{ijt}$ is a vector of control variables, including the logarithm of household income, the logarithm of household total debts, family size, individual’s age, marriage status, health condition, education, and social status. $u_{j}$ and $\lambda_{t}$ are household fixed effects and year fixed effects, respectively. Standard errors are clustered at household level. The parameter β is of interest in this study. If the TPA program helps to reduce individuals’ depression, the parameter β should have a negative sign with statistical significance.

## Results

### Main Results

Table 3 displays the estimation results of Equation 1, with column (1) excluding control variables and column (2) including them. As shown in both columns, the coefficients of Treat×Post are negative and statistically significant at the 1% level, indicating that the TPA program could reduce individuals’ depression. According to the point estimate in column (2), the TPA program reduces depression by 0.116 points, which accounts for 6.82% of the standard deviation of depression scores. Given that many of the poor households are in rural areas, column (3) reports the estimation results for the rural sample. The findings in column (3) align with those in columns (1) and (2), reinforcing the robustness of our main results. Overall, the results indicates that the TPA program contributes to alleviating depression among affected individuals.

**TABLE 3:** Effect of Targeted Poverty Alleviation program on depression (China, 2024).

	Depression		
	Full sample (1)	Full sample (2)	Rural sample (3)
*Treat* × *Post*	−0.1324^***^	−0.1160^***^	−0.1635^***^
	(0.0310)	(0.0295)	(0.0380)
*Log(Income)*		−0.0586^***^	−0.0685^***^
		(0.0070)	(0.0097)
*Log(Debts)*		0.0102^***^	0.0106^***^
		(0.0012)	(0.0019)
*Family size*		−0.0087	0.0021
		(0.0054)	(0.0078)
*Age*		−0.0025^***^	0.0008
		(0.0006)	(0.0007)
*Marriage*		−0.1812^***^	−0.1992^***^
		(0.0167)	(0.0237)
*Health*		−0.3696^***^	−0.3541^***^
		(0.0053)	(0.0072)
*Education*		−0.0347^***^	−0.0425^***^
		(0.0020)	(0.0026)
*Social status*		−0.0969^***^	−0.0785^***^
		(0.0057)	(0.0078)
Constant	2.2000^***^	4.7281^***^	4.6895^***^
	(0.0032)	(0.0836)	(0.1119)
Year fixed effects	Yes	Yes	Yes
Household fixed effects	Yes	Yes	Yes
Observations	129,713	129,713	65,138
Adj. R2	0.2339	0.2961	0.3010

This table reports the results of the DID model in Equation 1. The dependent variable is the depression level. Standard errors clustered by household are reported in parentheses. ^*^, ^**^, and ^***^ indicate statistical significance at the 10%, 5%, and 1% levels, respectively.

Our results are robust to (1) changing the definition of treatment group, (2) correcting sample selection bias with propensity score matching (PSM) approach. In addition, our DID model also satisfied the basic assumption of common trend. Finally, we conduct a placebo test to exclude that our findings are not driven by other unobserved or omitted factors. See more details about the robustness checks in Supplementary File S2.

### Mechanisms Analyses

In this subsection, we try to explore four mechanisms through which the TPA program may affect individuals’ depression levels: (1) by improving local medical conditions, (2) by cutting down household healthcare spending, (3) by increasing household entertainment expenses, and (4) by increasing the likelihood living in family.

To conduct mechanisms analyses, we set following mediating model [20]:

$$M_{ijt}=\alpha +\beta Treat_{j}\times Post_{t}+\gamma X_{ijt}+u_{j}+\lambda_{t}+\varepsilon_{ijt}$$ (2)

$$Depression_{ijt}=\alpha +\beta Treat_{j}\times Post_{t}+\theta M_{ijt}+\gamma X_{ijt}+u_{j}+\lambda_{t}+\varepsilon_{ijt}$$ (3)

where $M_{ijt}$ is the mediator variable, including medical condition, healthcare spending, entertainment expenses, and living in family. Other variables are the same as in Equation 1.

If $M_{ijt}$ is a valid mechanism, we expect the coefficient of $Treat_{j}\times Post_{t}$ is significant in Equation 2, suggesting that the TPA program could affect the mediator variable $M_{ijt}$ . Moreover, we also expect that the coefficient of $M_{ijt}$ is significant in Equation 3, indicating that $M_{ijt}$ could also influence individuals’ depressive level.

#### Improving Medical Conditions

Medical conditions indeed play a crucial role in influencing individuals’ depressive symptoms. Improved medical infrastructure and access to healthcare services can enhance the identification and management of mental health issues. This improvement enables healthcare professionals to deploy more effective interventions promptly, potentially alleviating symptoms more efficiently.

Moreover, enhanced medical conditions contribute to better treatment outcomes for individuals suffering from illnesses and diseases. Effective treatment reduces anxiety associated with health concerns, thereby potentially decreasing overall levels of depression among affected individuals.

Therefore, if the Targeted Poverty Alleviation (TPA) program succeeds in enhancing medical conditions, it could potentially lead to a reduction in individuals’ depression. This underscores the broader impact that comprehensive poverty alleviation strategies can have on mental health outcomes through improved healthcare access and quality.

Given that the actual medical conditions are difficult to measure, we use individuals’ perceptions of the local medical level as a proxy of local medical conditions. For measurement of local medical conditions, the CFPS survey asked respondents to answer “What do you think of the level of medical expertise there? 1. Very bad. 2. Bad. 3. Fair. 4. Good. 5. Very good.” Then, we estimate Equations 2, 3. The estimation results are shown in columns (1) and (2) of Table 4. We can find that the coefficient of Treat×Post is positive and significant at 1% in column (1), suggesting that the TPA program indeed improves local medical conditions. Moreover, the coefficient of medical level is significantly negative in column (2), indicating that medical conditions are negatively related to levels of depression. Thus, improving medical conditions is a valid mechanism through which the TPA program reduces individuals’ depression.

**TABLE 4:** Mechanism analyses (China, 2024).

Mechanisms	Medical conditions		Household healthcare spending		Household entertainment expenses		Living in family or not	
	Medical level (1)	Depression (2)	Healthcare spending (3)	Depression (4)	Entertainment expenses (5)	Depression (6)	Living in family (7)	Depression (8)
*Treat* × *Post*	0.1160^***^	−0.0978^***^	−0.1063^*^	−0.1105^***^	0.2917^***^	−0.1001^***^	0.0124^***^	−0.1066^***^
	(0.0295)	(0.0296)	(0.0575)	(0.0296)	(0.0572)	(0.0296)	(0.0039)	(0.0295)
*Medical level*		−0.0467^***^						
		(0.0069)						
*Log(Healthcare spending)*				0.0141^***^				
				(0.0024)				
*Log(Entertainment expenses)*						−0.0122^***^		
						(0.0020)		
*Living in family*								−0.0506^**^
								(0.0235)
Controls	Yes	Yes	Yes	Yes	Yes	Yes	Yes	Yes
Year fixed effects	Yes	Yes	Yes	Yes	Yes	Yes	Yes	Yes
Household fixed effects	Yes	Yes	Yes	Yes	Yes	Yes	Yes	Yes
Observations	129,713	129,150	53,785	129,016	53,951	129,317	129,713	129,713
Adj. R2	0.2961	0.2965	0.2517	0.2960	0.4853	0.2964	0.0752	0.2961

This table explores whether the TPA program affects depression through following channels: medial conditions, household healthcare spending, household entertainment expenses, and living in family or not. In columns (3) and (5), the control variables include household characteristics and household head’s characteristics, whereas the control variables in other columns include household-level and individual-level characteristics. Standard errors clustered by household are reported in parentheses. ^*^, ^**^, and ^***^ indicate statistical significance at the 10%, 5%, and 1% levels, respectively.

#### Cutting Down Household Healthcare Spending

In impoverished families, healthcare expenditures often represent a significant financial burden, contributing to heightened levels of depression and anxiety [8]. The Targeted Poverty Alleviation (TPA) program facilitated reforms in healthcare and extended social security coverage to include poor households, potentially alleviating this economic strain. By analyzing household healthcare spending data and employing Equations 2, 3 to assess the mediating effect, we examine whether reduced healthcare spending serves as a viable mechanism.

In CFPS survey, there are two questions: (1) In the past year, the total direct medical expenditure (excluding that was reimbursed or reimbursable but including that was paid by or borrowed from relatives) was ___________ Yuan; (2) In the past year, the total expenditure on fitness (e.g., bodybuilding, physical exercise, and health-related apparatus and products) as ________ Yuan. We use the sum of the two questions’ values to measure household healthcare spending. As demonstrated in column (3) of Table 4, we find a significantly negative coefficient for Treat×Post , indicating that the TPA program effectively reduced household healthcare expenditures. Furthermore, the positive coefficient for Log (Healthcare spending) suggests that higher healthcare spending correlates with increased levels of depression. Therefore, the observed reduction in depression associated with the TPA program can be attributed to its impact on lowering household healthcare costs.

These findings underscore the TPA program’s role in mitigating individuals’ depression by alleviating financial pressures related to healthcare expenditures within poor households.

#### Increasing Household Entertainment Expenses

The Targeted Poverty Alleviation (TPA) program has been shown to increase rural household income and consumption [6], which includes expenditures on household entertainment. Research indicates that engagement in entertainment activities and travel contributes positively to mental health [21, 22]. Therefore, household entertainment expenses represent a plausible mechanism through which the TPA program may alleviate depression.

In CFPS survey, there are two questions: (1) In the past month, the total expenditure on entertainment (including purchasing books, newspapers, magazines, VCDs, and DVDs, and going to cinemas and bars, and so on) was _____Yuan; (2) In the past year, the total expenditure on tourism was _____ Yuan. The household entertainment expenses are measured by 10 times value in question (1) plus value in question (2). The mechanism check results are presented in columns (5) and (6) of Table 4. Column (5) reveals a significant increase in household entertainment expenses attributable to the TPA program, while column (6) demonstrates a negative association between household entertainment expenditures and individuals’ depression levels. These empirical findings provide support for the hypothesis that augmenting household entertainment expenses serves as a viable pathway through which the TPA program reduces depression.

#### Increasing the Likelihood Living in Family

Research indicates that living with family is associated with increased family happiness and lower levels of depression [10]. Therefore, if the Targeted Poverty Alleviation (TPA) program increases the likelihood of individuals living with their families, it could potentially reduce depression. The TPA program focused on poverty alleviation through industrial development, which generated local employment opportunities and encouraged individuals to remain in their hometowns. Additionally, infrastructure improvements supported by the TPA program, such as enhanced transportation networks, facilitated individuals living with their families.

For measurement of living in family, the CFPS survey asked to answer “You have just mentioned that “Load the name of each family member” is “not/still” living in this family. Please confirm again that whether or not he/she is living in this family. Short-term absence, i.e., the person will return within 3 months, is treated as living in this family. 1. Yes. 0. No.” We estimate the mediating model using Equations 2, 3, with results presented in columns (7) and (8) of Table 4. Column (7) demonstrates that the TPA program increases the probability of individuals living with their families. Column (8) shows a negative relationship between living with family and depression levels. These findings provide empirical evidence supporting the hypothesis that increasing the likelihood of individuals living with their families is a valid mechanism through which the TPA program reduces depression.

## Discussion

This study investigates the impact of the Targeted Poverty Alleviation (TPA) program on mitigating depression and explores the mechanisms through which it affects individuals’ mental health. Using data from the China Family Panel Studies (CFPS) survey spanning 2012 to 2020, we employ a Difference-in-Difference (DID) model to estimate these effects. Our findings reveal that the TPA program significantly reduces individuals’ levels of depression. Specifically, the program lowers depression scores by 0.116 points, which represents a 6.82% decrease relative to the standard deviation of depression scores.

Furthermore, the mechanism analyses indicate that the TPA program influences individuals’ depression levels through several channels: (1) improving local medical conditions, (2) reducing household healthcare spending, (3) increasing household entertainment expenses, and (4) enhancing the likelihood of living in family.

Our results have important implications for policymakers who aim to improve the poor’s mental health. Policymakers should incorporate medical conditions, poor households’ healthcare spending, household entertainment, and likelihood to live with family into the poverty alleviation policies so that the policies can better serve the poor by improving their mental health. For example, China implements some local employment programs (such 以工代赈). This kind of programs can increase local employment and income. They can also enable the poor to have more time living with their family and improve family happiness. In addition, the governments can initiate programs which constructing entertainment facilities. This kind of programs can not only provide local employment opportunities, but also increase entertainment activities, which ultimately decreases depression.

There are two limitations in our study. First, although we explore various channels through which the TPA program affects depression levels, it is worth to note the possibility of reverse causality or omitted variable bias in the mediation analysis. Future research can be devoted to investigate between these channels and depression. Second, we examine the overall impact of TPA on depression. However, TPA program has five batches, including industrial development, relocation, eco-compensation, education, and social security. Different batches may have heterogeneous effects on depression. Future research can explore how each batch affects individuals’ depression level.
