# Supplementary material for: Can Targeted Poverty Alleviation Program Reduce Depression? Evidence From China
Source: Int J Public Health. 2024 Aug 30;69:1607106. doi: 10.3389/ijph.2024.1607106 (PMC11392683; doi:10.3389/ijph.2024.1607106)
Supplement: Supplementary file 1 [file DataSheet2.docx]

**1. Robustness checks**

**1.1 Changing the definition of treatment group**

In our primary analysis, the treatment variable $Treat$ is defined as 1 if an individual’s household income per capita is below the poverty line in either 2014 CFPS or 2016 CFPS, and 0 otherwise. However, the implementation of the TPA program may suffer from Errors of inclusion (households identified as poor that did not need assistance) and exclusion (poor households that failed to receive adequate support), because the government lacked reliable income data. Similarly, the income data in CFPS might also have measurement errors. To reinforce the validity of previous findings, we try four alternative ways to define treatment status. (1) The first treatment variable $Treat1$ is defined as 1 if a household income per capita is below the poverty line in 2014 CFPS, and 0 otherwise. (2) The second treatment variable $Treat2$ is defined as 1 if a household income per capita is below the poverty line in either 2012 CFPS or 2014 CFPS, and 0 otherwise. (3) The third treatment variable $Treat3$ is defined as 1 if a household income per capita are below the poverty lines in both 2014 CFPS and 2016 CFPS, and 0 other wise. (4) The fourth treatment variable $Treat4$ is defined as 1 if the household head said that household had poverty registration in 2020 CFPS, and 0 otherwise.

We use $Treat1$, $Treat2$, $Treat3$, and $Treat4$ to re-run equation (1). The estimation results are shown in Table A1. As shown in Table A1, we can find that the results are still consistent with our main finding that TPA program helps reduce depression.

**<Table A1 insert here>**

**1.2 Propensity score matching (PSM) – DID**

Our main findings may also suffer from sample selection bias because households included in the TPA program may be quite different from those not included in the program. Thus, we use propensity score matching (PSM) approach to address this concern.

Specifically, we first run a logistic regression to estimate the probability of a household to be included in the program on the following variables: the logarithm of household income, the logarithm of household total assets, the logarithm of household total debts, family size, household head’s age, marriage status, health condition, education, and social status. Then, we match individuals based on the propensity score. To validate the robustness, we use radius matching approach, kernel matching approach, and 1 to 4 matching approach, respectively. Finally, we re-run equation (1) with the matched samples. The results are shown in Table A2. The regression outcomes indicate that TPA program decreases individuals’ depression symptoms. Thus, our main findings are valid.

**<Table A2 insert here>**

**1.3 Testing common trend of DID model**

A basic assumption of the DID model is that treatment group and control group should have common trend in pre-intervention period. To examine this assumption, we first run the following model: $Depression_{ijt}=\alpha+\beta_{1}Treat_{i}\times Y2012+\beta_{2}Treat_{i}\times Y2016+\beta_{3}Treat_{i}\times Y2018+\beta_{4}Treat_{i}\times Y2020+\boldsymbol{\gamma}\boldsymbol{X}_{ijt}+u_{j}+\lambda_{t}+\varepsilon_{ijt}$. Here, $Y2012$ to $Y2020$ are year dummies for 2012, 2016, 2018, and 2020, respectively. We use 2014 as baseline. Then, we plot the estimates $\beta_{1}$ to $\beta_{4}$ and associated 90% confidence intervals in Figure A1. We observe that the coefficients are all significant after 2015. Furthermore, we find no significant trend between treatment group and control group before the TPA program.

**<Figure A1 insert here>**

**1.4 Placebo test**

To further validate the credibility of our results, we conduct a placebo test. First, we randomly assign the treatment status, i.e., placebo $Treat$. Second, we re-run equation (1) with placebo $Treat\times Post$. Finally, we repeat the simulation 1,000 times and plot the distribution of the coefficients of placebo $Treat\times Post$ in Figure A2. For comparison, in Figure A2, we also add a line for the actual coefficient of $Treat\times Post$ in column (2) of Table 3. From Figure A2, we find that the actual line lies far away from the placebo coefficients. It suggests that our findings are not driven by other unobserved or omitted factors.

**<Figure A2 insert here>**


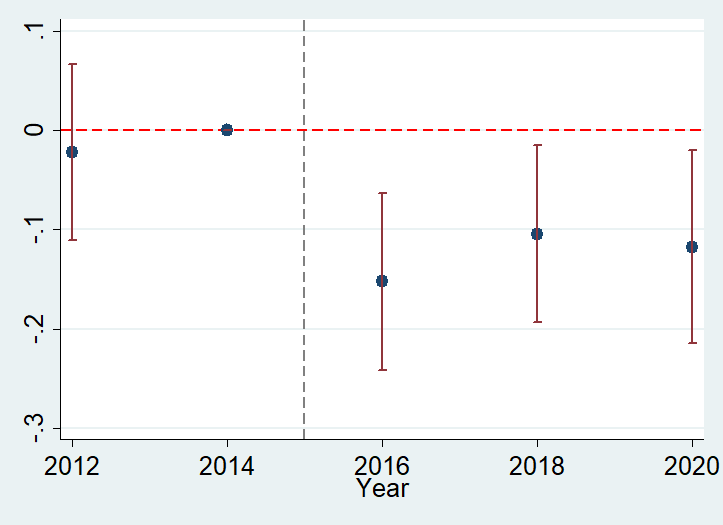


Figure A1 The effect of Targeted Poverty Alleviation program on depression over years (China, 2024)

Note: The figure displays the coefficients ($\beta_{1}$ to $\beta_{4}$) and associated 90% confidence intervals from estimating the following model: $Depression_{ijt}=\alpha+\beta_{1}Treat_{i}\times Y2012+\beta_{2}Treat_{i}\times Y2016+\beta_{3}Treat_{i}\times Y2018+\beta_{4}Treat_{i}\times Y2020+\boldsymbol{\gamma}\boldsymbol{X}_{ijt}+u_{j}+\lambda_{t}+\varepsilon_{ijt}$. We use 2014 as baseline.


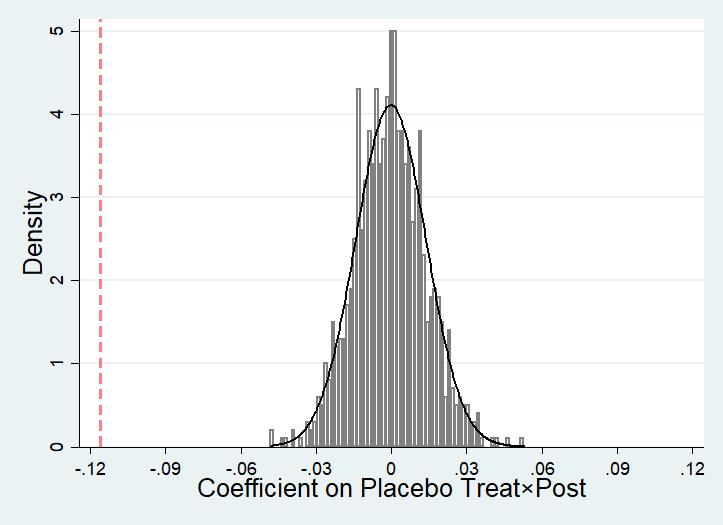


Figure 2 Placebo test: Random assignment of treatment status (China, 2024)

Note: This figure reports results on the impact of TPA program on depression based on random assignment of treatment status. We randomly assign the treatment status and then re-estimate equation (1) by replace $Treat\times Post$ with placebo $Treat\times Post$. We repeat the simulation 1000 times and plot the distribution of the placebo coefficients. The red line is the coefficient of $Treat\times Post$ in column (2) of Table 3.Table A1 Robustness check: Changing the definition of treatment status (China, 2024)

This table reports the results of equation (1) when dependent variable is replaced with four alternative measures of treatment status. In column (1), $Treat1$ is defined as 1 if a household income per capita is below the poverty line in 2014 CFPS, and 0 otherwise. In column (2), $Treat2$ is defined as 1 if a household income per capita is below the poverty line in either 2012 CFPS or 2014 CFPS, and 0 otherwise. In column (3), $Treat3$ is defined as 1 if a household income per capita are below the poverty lines in both 2014 CFPS and 2016 CFPS, and 0 other wise. In column (4), $Treat4$ is defined as 1 if the household head said that household had poverty registration in 2020 CFPS, and 0 otherwise. Standard errors clustered by household are reported in parentheses. ^*^, ^**^, and ^***^ indicate statistical significance at the 10%, 5%, and 1% levels, respectively.

|  | Depression | | | |
| --- | --- | --- | --- | --- |
|  | (1) | (2) | (3) | (4) |
| *Treat1*$\boldsymbol{\times}$*Post* | -0.1264^***^ |  |  |  |
|  | (0.0337) |  |  |  |
| *Treat2*$\boldsymbol{\times}$*Post* |  | -0.0717^***^ |  |  |
|  |  | (0.0268) |  |  |
| *Treat3*$\boldsymbol{\times}$*Post* |  |  | -0.1819^**^ |  |
|  |  |  | (0.0767) |  |
| *Treat4*$\boldsymbol{\times}$*Post* |  |  |  | -0.1066^**^ |
|  |  |  |  | (0.0454) |
| *Log(Income)* | -0.0569^***^ | -0.0552^***^ | -0.0586^***^ | -0.0589^***^ |
|  | (0.0070) | (0.0071) | (0.0070) | (0.0070) |
| *Log(Debts)* | 0.0102^***^ | 0.0102^***^ | 0.0102^***^ | 0.0103^***^ |
|  | (0.0012) | (0.0012) | (0.0012) | (0.0012) |
| *Family size* | -0.0086 | -0.0089 | -0.0086 | -0.0090* |
|  | (0.0054) | (0.0054) | (0.0054) | (0.0054) |
| *Age* | -0.0025^***^ | -0.0025^***^ | -0.0025^***^ | -0.0025^***^ |
|  | (0.0006) | (0.0006) | (0.0006) | (0.0006) |
| *Marriage* | -0.1810^***^ | -0.1813^***^ | -0.1805^***^ | -0.1805^***^ |
|  | (0.0167) | (0.0167) | (0.0167) | (0.0167) |
| *Health* | -0.3697^***^ | -0.3697^***^ | -0.3697^***^ | -0.3696^***^ |
|  | (0.0053) | (0.0053) | (0.0053) | (0.0053) |
| *Education* | -0.0347^***^ | -0.0348^***^ | -0.0348^***^ | -0.0347^***^ |
|  | (0.0020) | (0.0020) | (0.0020) | (0.0020) |
| *Social status* | -0.0969^***^ | -0.0970^***^ | -0.0971^***^ | -0.0970^***^ |
|  | (0.0057) | (0.0057) | (0.0057) | (0.0057) |
| Constant | 4.7072^***^ | 4.6908^***^ | 4.7192^***^ | 4.7247^***^ |
|  | (0.0835) | (0.0842) | (0.0836) | (0.0835) |
| Year fixed effects | Yes | Yes | Yes | Yes |
| Household fixed effects | Yes | Yes | Yes | Yes |
| Observations | 129713 | 129713 | 129713 | 129713 |
| Adj. R2 | 0.2961 | 0.2960 | 0.2960 | 0.2960 |

Table A2 Robustness check: Propensity score matching (PSM) (China, 2024)

This table reports the results of the PSM-DID. We first run a logistic model to estimate the propensity score and then match samples with radius matching approach, kernel matching approach, and 1 to 4 matching approach, respectively. Finally, we re-run equation (1) with the matched samples. Standard errors clustered by household are reported in parentheses. ^*^, ^**^, and ^***^ indicate statistical significance at the 10%, 5%, and 1% levels, respectively.

|  | Depression | | |
| --- | --- | --- | --- |
|  | Radius Matching  (1) | Kernel Matching  (2) | 1 to 4 Matching  (3) |
| *Treat*$\boldsymbol{\times}$*Post* | -0.1112^***^ | -0.1104^***^ | -0.0913^***^ |
|  | (0.0307) | (0.0307) | (0.0343) |
| *Log(Income)* | -0.0557^***^ | -0.0552^***^ | -0.0578^***^ |
|  | (0.0074) | (0.0074) | (0.0098) |
| *Log(Debts)* | 0.0109^***^ | 0.0109^***^ | 0.0107^***^ |
|  | (0.0013) | (0.0013) | (0.0019) |
| *Family size* | -0.0055 | -0.0055 | -0.0057 |
|  | (0.0058) | (0.0058) | (0.0079) |
| *Age* | -0.0027^***^ | -0.0027^***^ | 0.0001 |
|  | (0.0006) | (0.0006) | (0.0008) |
| *Marriage* | -0.1852^***^ | -0.1853^***^ | -0.1976^***^ |
|  | (0.0179) | (0.0179) | (0.0257) |
| *Health* | -0.3720^***^ | -0.3718^***^ | -0.3821^***^ |
|  | (0.0056) | (0.0056) | (0.0078) |
| *Education* | -0.0347^***^ | -0.0347^***^ | -0.0369^***^ |
|  | (0.0021) | (0.0021) | (0.0030) |
| *Social status* | -0.0943^***^ | -0.0943^***^ | -0.0904^***^ |
|  | (0.0060) | (0.0060) | (0.0084) |
| Constant | 4.6849^***^ | 4.6799^***^ | 4.7280^***^ |
|  | (0.0889) | (0.0890) | (0.1164) |
| Year fixed effects | Yes | Yes | Yes |
| Household fixed effects | Yes | Yes | Yes |
| Observations | 115,799 | 115,812 | 59,135 |
| Adj. R2 | 0.2930 | 0.2930 | 0.2993 |
