# Supplementary material for: Can Targeted Poverty Alleviation Program Reduce Depression? Evidence From China
Source: Int J Public Health. 2024 Aug 30;69:1607106. doi: 10.3389/ijph.2024.1607106 (PMC11392683; doi:10.3389/ijph.2024.1607106)
Supplement: Supplementary file 2 [file DataSheet1.docx]

1. **Institutional background**

China has a rich history of combating poverty, spanning distinct stages from its founding in 1949 up to 2012. This progression can be categorized into five main stages.

The first stage, lasting from 1949 to 1977, focused on relief-type poverty alleviation. During this period, most rural households in China endured extreme poverty. Government efforts primarily aimed at ensuring the basic survival of rural populations through low-level social assistance, including mutual aid and cooperative initiatives [1].

The second stage, from 1978 to 1985, marked a shift towards structural reform-driven poverty relief. This phase saw the implementation of reforms such as rural land system reforms, market mechanism adjustments, and employment system reforms [1-2]. These reforms substantially boosted economic productivity and rapidly increased farmers' incomes, leading to a significant reduction in rural poverty.

The third stage, spanning from 1986 to 1993, was characterized by a development-oriented approach to poverty alleviation. Recognizing persistent poverty in certain regions, particularly in western rural areas, the government shifted its focus to the county level. It identified 331 poverty-stricken counties as specific targets for anti-poverty efforts.

The fourth stage, occurring from 1994 to 2000, concentrated on addressing critical challenges in poverty relief. During this period, the government launched the "Priority Poverty Alleviation Plan (1994-2000)," prioritizing basic needs such as food and clothing for impoverished populations in underdeveloped and backward regions.

The fifth stage, from 2001 to 2012, emphasized comprehensive consolidation-oriented poverty alleviation efforts. The "Outline of Development-driven Poverty Alleviation in China’s Rural Areas (2001-2010)" underscored the need for a more targeted approach, shifting the focus from counties to individual villages. As a result, 148 thousand impoverished villages were identified as primary targets for anti-poverty policies, marking a trend towards increasingly precise targeting of poverty alleviation efforts.

In 2013, General Secretary Xi Jinping introduced the innovative strategy of targeted poverty alleviation (TPA) during an inspection in Hunan province. This marked a pivotal shift in anti-poverty efforts from broad geographical levels (county and village) to precise household and individual scales. Subsequently, the government launched the "Opinions on Innovative Mechanisms to Promote Rural Poverty Alleviation and Development" in 2014, establishing the national "Jiandang Lika" database to register households with incomes below the national poverty line.

From 2015 onwards, China initiated the "Battle Against Extreme Poverty," committing to fully implement the TPA program with the ambitious goal of lifting all rural poor households out of poverty by 2020. This comprehensive initiative included six precise measures: accurate identification of beneficiaries, precise project planning, targeted fund allocation, effective implementation of aid measures, precise assignment of responsibility, and accurate assessment of poverty reduction outcomes [1]. Additionally, it encompassed five major initiatives: industrial development, relocation, ecological compensation, education enhancement, and social security improvements.

The success of the TPA program was bolstered by innovative approaches such as the development of "Internet Plus," enhancements in public services, and infrastructure construction. These efforts culminated in China achieving the monumental task of eradicating extreme poverty.^[[1]](#footnote-1)^

**References**

1. Guo Y, Zhou Y, Liu Y. Targeted poverty alleviation and its practices in rural China: A case study of Fuping county, Hebei Province. Journal of Rural Studies. 2022 Jul 1;93:430-40.

2. Zhu X, Peng C. 40 Years of China's War on Poverty. China Renmin University Press; 2022 Sep 3.

1. See more details at http://www.xinhuanet.com/english/2021-04/06/c_139860414.htm. [↑](#footnote-ref-1)
